# Gene mutation profiling in Chinese colorectal cancer patients and its association with clinicopathological characteristics and prognosis

**DOI:** 10.1002/cam4.2727

**Published:** 2019-11-28

**Authors:** Zu‐Lu Ye, Miao‐Zhen Qiu, Tao Tang, Fang Wang, Yi‐Xin Zhou, Meng‐Jie Lei, Wen‐Long Guan, Cai‐Yun He

**Affiliations:** ^1^ Department of Molecular Diagnostics State Key Laboratory of Oncology in South China Collaborative Innovation Center for Cancer Medicine Sun Yat‐Sen University Cancer Center Guangzhou China; ^2^ Department of Medical Oncology State Key Laboratory of Oncology in South China Collaborative Innovation Center for Cancer Medicine Sun Yat‐Sen University Cancer Center Guangzhou China; ^3^ Department of VIP State Key Laboratory of Oncology in South China Collaborative Innovation Center for Cancer Medicine Sun Yat‐Sen University Cancer Center Guangzhou China

**Keywords:** colorectal cancer, mutation profiling, prognosis

## Abstract

**Background:**

Gene mutations may play an important role in the development, response to treatment and prognosis of colorectal cancer (CRC). This retrospective study aimed to investigate the mutation profiling of Chinese patients with CRC, and its correlation with clinicopathological features and prognosis.

**Methods:**

This study included 1190 Chinese CRC patients who were diagnosed between May 1998 and December 2018 and received clinical genetic testing. The OncoCarta Panel was used to test a total of 238 possible mutations in 19 common oncogenes.

**Results:**

Five hundred and eighty‐two (48.9%) cases were detected with gene mutations. Of the 582 cases, there were 111 cases (19.7%) with two concurrent mutations, and six cases (1.0%) with three concurrent mutations. KRAS was the most common gene mutation that occurred in all cases (429, 36.1%), followed by PIK3CA (121, 10.2%), NRAS (47, 3.9%), BRAF (35, 2.9%), HRAS (11, 0.9%) and epidermal growth factor receptor (EGFR) (11, 0.9%). AKT1, KIT, FGFR1, FGFR3, FLT3, CDK, ERBB2, ABL1, MET, RET and PDGFRA mutations were also detected in several cases. When it came to prognosis, we found that KRAS/NRAS/PIK3CA/BRAF mutation was not associated with prognosis. But BRAF mutation was associated with poor prognosis in patients who accepted anti‐EGFR therapy.

**Conclusions:**

The molecular testing offered the clinical data and mutation profile of Chinese CRC patients. The information of these mutated genes may help to find out the correlation between mutated genes and the development or prognosis of CRC.

## Graphical abstract

This is the largest sample size from Chinese colorectal cancer patients about gene mutation profiling. We found that KRAS was the most common mutated gene, followed by PIK3CA, NRAS and BRAF genes. BRAF mutation was associated with poor prognosis in patients who accepted anti‐epidermal growth factor receptor therapy.

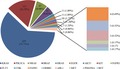

## 1 INTRODUCTION

Colorectal cancer (CRC) is the third most prevalent malignancy in the world. About 274.8 thousand new CRC and 132.1 thousand CRC‐related deaths occurred in China in 2010,^1^ and these two numbers are expected to be 624.3 and 221.1 thousand by 2025.^2^ Currently, tumor node metastasis (TNM) stage is the most important prognostic factor. However, even patients of the same stage may have different prognosis. Though CRC is considered a sporadic disease, it has been proved to be associated with genetic variants, including microsatellite instability (MSI), chromosomal instability, and RAS‐RAF‐MAPK mutation. It has been suggested that molecular biomarkers, such as KRAS, BRAF, PIK3CA, may have prognostic value in CRC.^3^

Previous studies have identified improved outcome with addition of anti‐epidermal growth factor receptor (EGFR) therapy to chemotherapy in CRC patients with RAS wild type.^4^, ^5^ However, mutation of RAS family or BRAF may activate the downstream RAS‐RAF‐MAPK pathway, which is independent of EGFR inhibition, and associated with resistance to anti‐EGFR therapy.^6^ Hence the mutation profiles may help to select candidates for optimal therapy. The genetic analysis of CRC performed by The Cancer Genome Atlas Network (TCGA) in 2012 has identified several gene alterations that may be targetable.^7^ More mutations which are less common are also reported in many researches, though their clinical value is still unclear.^8^, ^9^, ^10^

Recent advances in gene mutation of CRC and its potential prognostic and predictive value for diagnosis, classification and treatment have led us to investigate the genetic profile of Chinese CRC patients, describe the clinicopathological features and explore the association between prognosis and mutation status.

## 2 MATERIALS AND METHODS

### 2.1 Clinical data

We conducted a retrospective review of 1190 CRC patients diagnosed between May 1998 and December 2018 and received clinical genetic testing at Sun Yat‐sen University Cancer Center (Guangzhou, China). All the patients were diagnosed as CRC by hematoxylin and eosin staining and histologically analysis. Clinic records, including sex, age, primary tumor site, histological type, grade, TNM stage at diagnosis, metastatic sites, family history, MSI/mismatch repair (MMR) status, date of diagnosis and date of last contact, were collected by the medical record system. We defined the primary tumor site as two categories: right‐sided (from cecum to transverse colon) and left‐sided (from spleen flexure to rectum). This study was performed in accordance with the Declaration of Helsinki protocols and was approved by the local Ethics Committee. All subjects gave written informed consent at their first visit.

### 2.2 Mutation detection

All tumor specimens for molecular analysis, including primary/metastatic samples from surgery, biopsies from endoscopy/puncture, were resected and sent to our center. Hematoxylin and eosin staining slides were reviewed by pathologists to select the area of most abundant tumor tissues. Sections (4‐6 μm) were cut and transferred to 1.5 mL Eppendorf tubes for DNA extraction. DNA was extracted using a QIAamp DNA FFPE Tissue Kit (Qiagen), according to the manufacturer's protocol. The quantity and quality of the isolated DNA was tested using a Nanodrop ND‐2000 Spectrophotometer (Thermo Scientific). The final DNA samples were diluted to 10 ng/μL for analysis.

The OncoCarta Panel version 1.0 (Sequenom Inc) was used for detection of a total of 238 possible mutations in 19 common oncogenes (Table S1). Twenty nanograms of DNA was amplified using 24 sets of OncoCarta polymerase chain reaction primers. Then an extension reaction was conducted based on the OncoCarta extension primers. After the salts were removed by using a cation exchange resin, the products were spotted onto a 384‐well SpectroChipII using the MassARRAY^®^ Nanodispenser RS1000 (Sequenom Inc) and analyzed on a MALDI‐TOF mass spectrometer (Sequenom Inc). High performance liquid chromatography purified water was used as the blank control, while normal human somatic cells were used as negative control in each experiment.

### 2.3 Statistical methods

The patients' clinicopathological features were summarized with descriptive statistics. Categorical variables were compared using Chi square test, and comparisons of continuous variables were performed using Student's *t* test. The 5‐year cause‐specific survival was calculated from the date of diagnosis to the date of cancer‐specific death. Survival among different variables was compared using Kaplan‐Meier estimates and the log‐rank test. Statistical analysis was carried out using the IBM SPSS Statistics 22.0.0 package software (SPSS Inc). All the P values were two‐sided, and statistical significance was set at *P* < .05.

## 3 RESULTS

### 3.1 Clinicopathological features

One thousand one hundred and ninety patients with CRC who received genetic testing were investigated. There were 283 (24.9%) right‐sided (cecum to transverse colon) and 901 (74.6%) left‐sided (splenic flexure to rectum) cases, the rest 6 patients had more than 1 primary tumor site, and could not be simply classified to either left‐sided or right‐sided. Most of the cases were diagnosed at a later stage (23.7% at stage III and 60.5% at stage IV). The most common metastatic site was the liver (638, 53.6%), followed by the lung (414, 34.8%), peritoneum (316, 26.6%), distant lymph nodes (264, 22.2%), and bones (97, 8.2%). Other metastatic sites include ovaries, spleen, adrenal glands, skeletal muscles, etc. Patients with KRAS mutation were more probable to be female, with right‐sided primary tumor location, well to moderated differentiation and lung metastasis. But these features were not seen in patients with NRAS mutations. BRAF mutation was associated with poor differentiation. BRAF^V600E^ mutation was associated with right‐sided location. None of these four genes (KRAS, NRAS, BRAF, PIK3CA) was associated with TNM stage or MSI status. The clinicopathological features are showed in Tables 1 and 2.

**Table 1:** Clinicopathological features of 1190 patients and their association with gene mutations

Clinicopathological features	N (%)	Mutation (%)									
		KRAS	*P*	NRAS	*P*	BRAF	*P*	PI3K	*P*	2 or more mutations	*P*
Age											
≤60	796 (66.9)	280(35.2)	.372	32 (4.0)	.859	23 (2.9)	.881	83 (10.4)	.674	72 (9.0)	.309
>60	394 (33.1)	149(37.8)		15 (3.8)		12 (3.0)		38 (9.6)		45 (11.4)	
Sex											
Male	756 (63.5)	245(32.4)	.001	31 (4.1)	.724	19 (2.5)	.249	71 (9.4)	.242	71 (9.4)	.001
Female	434 (36.5)	184 (42.4)		16 (3.7)		16 (3.7)		50 (11.5)		46 (10.6)	
Differentiation											
Well to moderate	783 (65.8)	301 (38.4)	.037	35 (4.5)	.327	16 (2.0)	.007	97 (12.4)	.003	93 (11.9)	.009
Poor or undifferentiated	315 (26.5)	100 (31.7)		10 (3.2)		16 (5.1)		20 (6.3)		20 (6.3)	
Unknown	92 (7.7)	28 (30.4)		2 (2.2)		3 (3.3)		4 (4.3)		4 (4.3)	
Histology											
Papillary/tubular adenocarcinoma	1045 (87.8）	381 (36.5)	.992	41 (3.9)	.51	30 (2.9)	.316	109 (10.4)	.14	108 (10.3)	.34
Mucinous adenocarcinoma/signet ring cell	67 (5.6)	24 (35.8)		1 (1.5)		4 (6.0)		2 (3.0)		2 (3.0)	
Mix	8 (0.7)	3 (37.5)		0		0		1 (12.5)		1 (12.5)	
Unknown	70 (5.9)	21 (30.0)		5 (7.1)		1 (1.4)		9 (12.9)		6 (8.6)	
Primary tumor location											
Right	283 (23.8)	129 (45.6)	<.001	9 (3.2)	.436	10 (3.5)	.511	41 (14.5)	.005	35 (12.4)	.001
Left	901 (75.7)	297 (33.0)		38 (4.2)		25 (2.8)		79 (8.8)		81 (9.0)	
Unknown	6 (0.5)	3 (50.0)		0		0		1 (16.7)		1 (16.7)	
Metastasis											
Liver	638 (53.6)	221 (34.6)	.229	22 (3.4)	.733	18 (2.8)	.811	63 (9.9)	.883	58 (9.1)	.764
Lung	414 (34.8)	183 (44.2)	<.001	12 (2.9)	.329	11 (2.7)	.683	34 (8.2)	.132	40 (9.7)	.023
Peritoneum	316 (26.6)	125 (39.6)	.143	6 (1.9)	.055	9 (2.8)	.921	33 (10.4)	.751	30 (9.5)	.935
Bone	97 (8.2)	32 (33)	.495	1 (1.0)	.154	3 (3.1)	.92	6 (6.2)	.192	6 (6.2)	.237
Distant lymph nodes	264 (22.2)	96 (36.4)	.947	12 (4.5)	.401	12 (4.5)	.074	25 (9.5)	.728	26 (9.8)	.654
Family history											
Yes	342 (28.7)	132 (38.6)	.234	13 (3.8)	.906	10 (2.9)	.951	37 (10.8)	.602	44 (12.9)	.082
No	836 (70.3)	292 (34.9)		33 (3.9)		25 (3.0)		82 (9.8)		72 (8.6)	
Unknown	12 (1.0)	5 (41.7)		1 (8.3)		0		2 (16.7)		1 (8.3)	
TNM stage											
I	20 (1.7)	8 (40.0)	.93	0	.489	0	.193	2 (10.0)	.991	1 (5.0)	.888
II	113 (9.5)	43 (38.1)		2 (1.8)		1 (0.9)		12 (10.6)		13 (11.5)	
III	282 (23.7)	101 (35.8)		12 (4.3)		13 (4.6)		27 (9.6)		31 (11.0)	
IV	720 (60.5)	255 (35.4)		30 (4.2)		21 (2.9)		70 (9.7)		66 (9.2)	
Unknown	55 (4.6)	22 (40.0)		3 (5.5)		0		10 (18.2)		6 (10.9)	
MSI											
MSS	139 (11.7)	51 (36.7)	.972	7 (5.0)	.676	3 (2.2)	.751	17 (12.2)	.382	15 (10.8)	.734
MSI‐L	14 (1.2)	5 (35.7)		1 (7.1)		0		1 (7.1)		1 (7.1)	
MSI‐H	12 (1.0)	4 (33.3)		0		0		0		0	
Unknown	1025 (86.1)	369 (36.0)		39 (3.8)		32 (3.1)		103 (10.0)		101 (9.9)	
MMR											
pMMR	382 (32.1)	148 (38.7)	.604	14 (3.7)	.163	12 (3.1)	.419	43 (11.3)	.301	49 (12.8)	.243
dMMR	35 (2.9)	12 (34.3)		3 (8.6)		2 (5.7)		6 (17.1)		4 (11.4)	
Unknown	773 (65.0)	269 (34.8)		30 (3.9)		21 (2.7)		72 (9.3)		64 (8.3)	

Abbreviation: MMR, mismatch repair; MSI, microsatellite instability; MSS, microsatellite stable; TNM, tumor node metastasis.

**Table 2:** Clinicopathological features of right and left‐sided CRC

Clinicopathological features	N (%)	Location		
		Right	Left	*P*
Age				
≤60	794 (67.0)	192 (67.8)	602 (66.8)	.748
>60	390 (32.9)	91 (32.2)	299 (33.2)	
Sex				
Male	753 (63.6)	155 (54.8)	598 (66.4)	<.001
Female	431 (36.4)	128 (45.2)	303 (33.6)	
Differentiation				
Well to moderate	779 (65.8)	163 (57.6)	616 (68.4)	.004
Poor or undifferentiated	315 (26.6)	93 (32.9)	222 (24.6)	
Unknown	90 (7.6)	27 (9.5)	63 (7.0)	
Histology				
Papillary/tubular adenocarcinoma	1041 (87.9）	248 (87.6)	793 (88.0)	.023
Mucinous adenocarcinoma/signet ring cell	67 (5.7)	24 (8.5)	43 (4.8)	
Mix	8 (0.7)	2 (0.7)	6 (0.7)	
Unknown	68 (5.7)	9 (3.2)	59 (6.5)	
Metastasis				
Liver	636 (53.7)	151 (53.4)	485 (53.8)	.964
Lung	412 (34.8)	84 (29.7)	328 (36.4)	.078
Peritoneum	315 (26.6)	115 (40.6)	200 (22.2)	<.001
Bone	96 (8.1)	12 (4.2)	84 (9.3)	.023
Distant lymph nodes	264 (22.3)	64 (22.6)	200 (22.2)	.758
Family history				
Yes	339 (28.6)	97 (34.3)	242 (26.9)	.053
No	833 (70.4)	183 (64.7)	650 (72.1)	
Unknown	12 (1.0)	5 (41.7)	9 (1.0)	
TNM stage				
I	20 (1.7)	4 (1.4)	16 (1.8)	.045
II	113 (9.5)	22 (7.8)	91 (10.1)	
III	281 (23.7)	61 (21.6)	220 (24.4)	
IV	716 (60.5)	190 (67.1)	526 (58.4)	
Unknown	54 (4.6)	6 (2.1)	48 (5.3)	
MSI				
MSS	139 (11.7)	39 (13.8)	100 (11.1)	.535
MSI‐L	14 (1.2)	3 (1.1)	11 (1.2)	
MSI‐H	12 (1.0)	4 (1.4)	8 (0.9)	
Unknown	1019 (86.1)	237 (83.7)	782 (86.8)	
MMR				
pMMR	382 (32.3)	100 (35.3)	282 (31.3)	.448
dMMR	35 (3.0)	8 (2.8)	27 (3.0)	
Unknown	767 (64.8)	175 (61.8)	592 (65.7)	
Mutation				
Wild type	605 (51.1)	117 (41.3)	488 (54.2)	.001
One mutated gene	463 (39.1)	131 (46.3)	332 (36.8))	
2 or more	116 (9.8)	35 (12.4)	81 (9.0)	

Abbreviations: CRC, colorectal cancer; MMR, mismatch repair; MSI, microsatellite instability; MSS, microsatellite stable; TNM, tumor node metastasis.

### 3.2 Mutation profiles and prognosis

**Figure 1:**
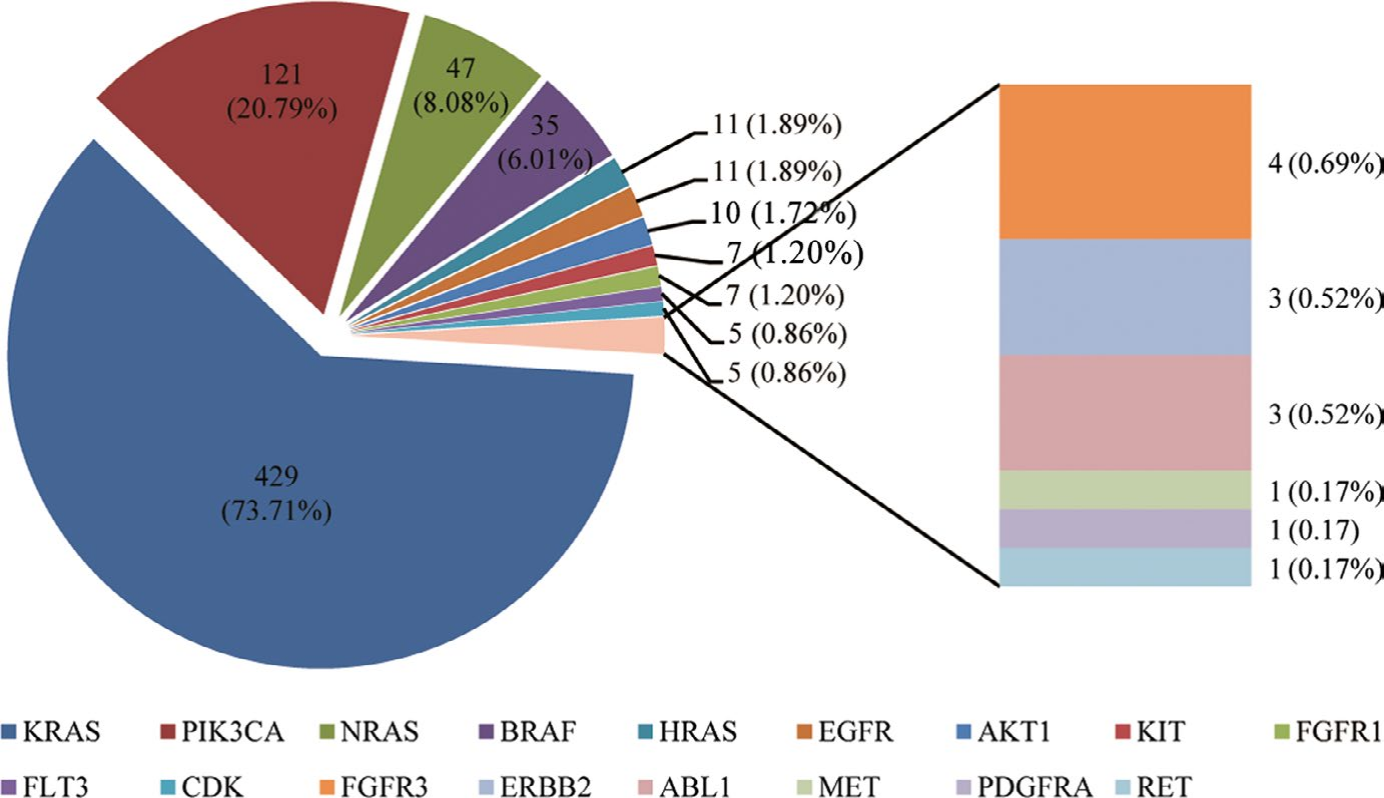
The frequency of different gene mutations in 582 patients with at least one mutation

**Figure 2:**
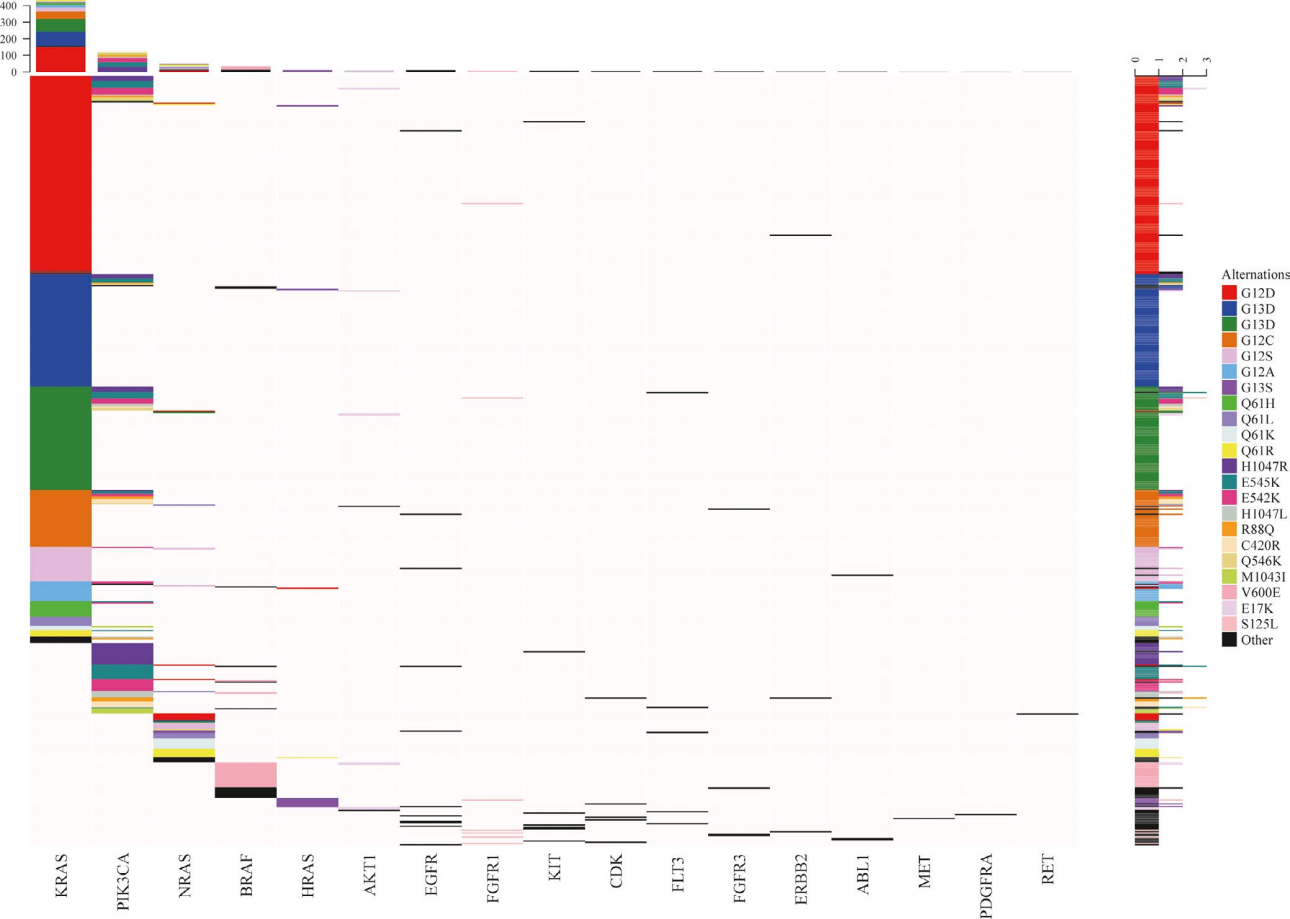
A schematic map of mutated genes in 582 patients with at least one mutation

**Figure 3:**
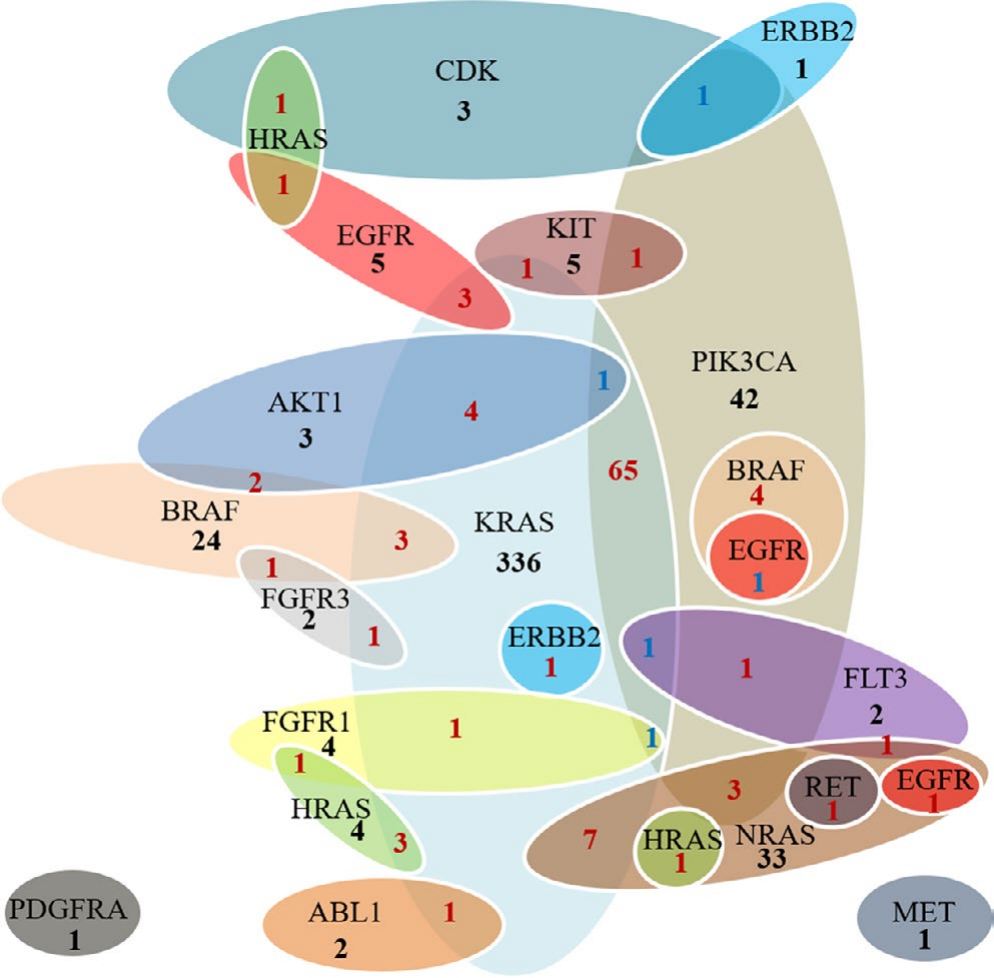
Association among different gene mutations

The mutations included in the OncoCarta panel are listed in Table S1. There were 582 (48.9%) cases with at least one gene mutation. All mutations detected are presented in Table S2. KRAS was the most common mutation in all cases (429, 36.1%), followed by PIK3CA (121, 10.2%), NRAS (47, 3.9%), BRAF (35, 2.9%), HRAS (11, 0.9%) and EGFR (11, 0.9%). AKT1, KIT, FGFR1, FGFR3, FLT3, CDK, ERBB2, ABL1, MET, RET and PDGFRA mutations were also detected in several cases (Figure 1). Of the 582 cases, there were 111 cases (19.7%) with two concurrent mutations, and six cases (1.0%) with three concurrent mutations. Some genes like KRAS/NRAS and BRAF are thought to be mutually exclusive as reported in previous studies.^7^, ^11^, ^12^ However we found three cases with KRAS and BRAF concurrent mutations in this study. A schematic map of the patients with at least one gene mutation in any of the listed genes above is shown in Figure 2, and the concomitant and exclusive relationship among these genes is visualized in Figure 3.

#### 3.2.1 RAS family

**Figure 4:**
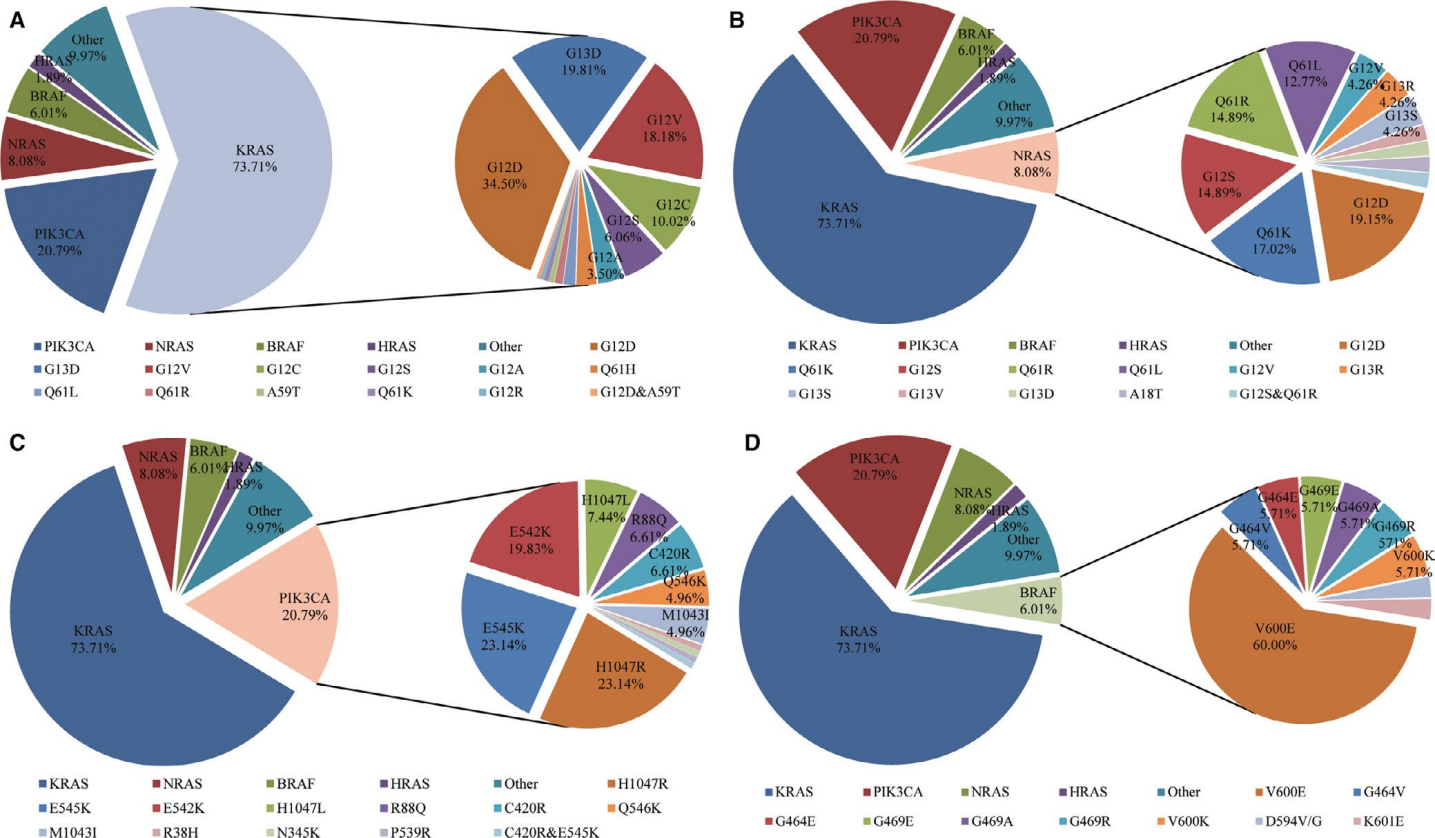
The distribution of mutation subtypes in KRAS (A), NRAS (B), PIK3CA (C) and BRAF (D)

There were 476 cases with at least one RAS gene mutation in our study (Table S3). KRAS mutation was the most frequently seen in codon 12 (72.7%) (Figure 4A). Two cases had double mutations in codon 12 and codon 59 in KRAS gene (G12D and A59T). The most frequent mutation occurred in codon 12 and codon 61 for NRAS (Figure 4B). One case was detected with double mutations in these two codons (G12S and Q61R). There were seven cases detected with both KRAS and NRAS mutations (G12A and G12S, G12C and Q61L, G12S and G12S, G12D and G12D, G12D and Q61R, G12V and G12D, G12V and G12V, exactly). Mutation was less commonly seen in HRAS gene (11 cases), and more frequently occurred in codon 13 (G13S, 81.8%). There were three cases with concurrent KRAS and HRAS mutations, which were G13D and G13S, G12A and G12D, G12D and G13S. One case was found have NRAS and HRAS comutation, which was G13R and Q61R (Figure 3).

**Figure 5:**
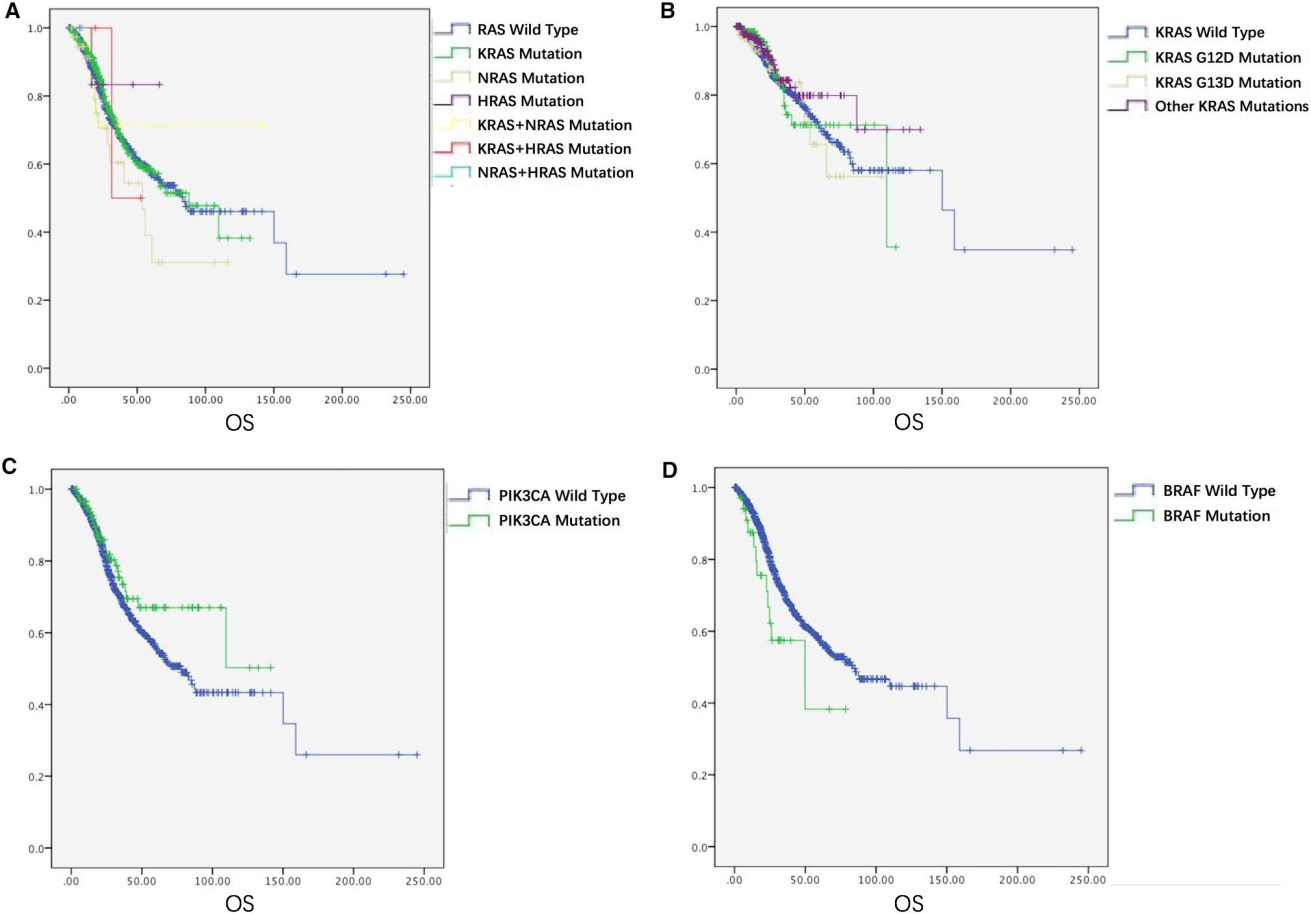
The relationship between gene mutation and prognosis of CRC patients. KRAS, NRAS and HRAS mutation (A). KRAS G12D and G13D mutation (B). PIK3CA mutation (C). BRAF mutation (D)

There was no significant difference of overall survival among different RAS mutation groups (Figure 5A,B). The median OS of RAS mutation was comparable to those cases with no RAS mutation (87.9 vs 82.9 months; *P* = .611).

#### 3.2.2 PIK3CA

PIK3CA gene mutation was the second most frequently seen in our study (121, 10.2%) (Figure 4C). Seventy nine cases (79/121, 65.3%) had not only PIK3CA mutaion, but also other gene mutations. PIK3CA‐KRAS was the most common comutation (68/79, 86.1%), which occurred in different codons of KRAS. PIK3CA exon 9 mutations were more significantly related with KRAS mutation (42/67, 62.7% vs 361/1069, 33.8% of wild type; *P* < .001). However, this association was not seen in exon 20 (18/43, 41.9%; *P* = .272). PIK3CA mutation was not related to prognosis of CRC (*P* = .115) (Figure 5C). Moreover, the survival of patients with PIK3CA‐KRAS mutation was comparable to patients with only PIK3CA/KRAS mutation (109.6 months vs not reached/70.7 months; *P* = .353 and .704, respectively).

#### 3.2.3 BRAF

BRAF gene mutation accounted for 6.0% (35/582) of all cases with mutation, BRAF^V600E^ mutation was most common (60%) (Figure 4D). Notably, we found three cases with BRAF and KRAS comutation (G12A and G464E, G13D and G464E, G13D and G469R). The prognosis of patients with BRAF mutation was much poorer than patients with other gene mutations or wild type tumors, though no statistical difference was reached (49.9 m vs 83.2 m, *P* = .051) (Figure 5D). Moreover, worse prognosis was found in patients with BRAF V600E mutation, compared to patients without BRAF mutation (24.8 m vs 83.2 m; *P* = .005).

#### 3.2.4 Prognosis according to diagnostic time, location and anti‐EGFR therapy

As no significant effect of gene mutation on prognosis of CRC was found, we tried to figure out if the prognosis was affected by diagnostic time, tumor locations or anti‐EGFR treatment. Since clinical practice has changed significantly in the last 5 years, we screened out the patients diagnosed before 2014. However, still no significant difference of prognosis was found in KRAS/NARS/PIK3CA/BRAF mutated patients, compared to those without these mutations (*P* = .197, .314, .683 and .191, respectively) (Figure S1). When it comes to primary sites, we found that the OS of right‐sided CRC (RCRC) patients was poorer than the left‐sided ones in KRAS mutation group (47.5 vs 87.9 months), though the result was not statistically significant (*P* = .157). However, in the group without KRAS mutation, the prognosis of RCRC patients was worse than the left‐sided ones (55.8 vs 85.3 months; *P* = .003). However, this phenomenon was not seen in NRAS/BRAF/PIK3CA mutation. Besides, in patients with specific sites of metastasis (liver, lung, peritoneum, bones, distant lymphnodes), no remarkable difference of prognosis was seen in the KRAS/NRAS/PIK3CA/BRAF mutated group compared to the wild type group. In patients who did not accept anti‐EGFR therapy, there was a difference of OS between patients with and without KRAS mutation, though it was not statistically remarkable (109.6 vs 65.1 months; *P* = .331) (Figure S2). On the other hand, in the anti‐EGFR therapy group, patients with BRAF mutation showed a poorer outcome than ones without BRAF mutation (15.9 vs 59.1 month; *P* = .043) (Figure S3).

## 4 DISCUSSION

As a pathologically and clinically heterogeneous disease, CRC presents different clinical features, treatment response and prognosis. Hence it is necessary to find out clinical or molecular markers which may have prognostic or predictive value. Several researches have analyzed the genetic profiling of CRC patients.^8^, ^9^, ^13^ In this study, we investigated 1190 Chinese CRC patients' mutation distribution pictures.

The primary tumor location of CRC has been emphasized over the recent years, because it may guide the treatment options according to several researches.^14^, ^15^ However, the association between primary site and gene mutation is not well investigated. In our study, RCRC is less common than left‐sided CRC (LCRC). Older age, female, mucinous adenocarcinoma, poor differentiation, advanced TNM stage and peritoneal metastasis are more commonly seen in RCRC (Table 2), which is consistent with previous reports.^16^ Some studies have showed that RCRC is associated with hypermutated and MSI‐high status. Besides, RAS, BRAF and PIK3CA mutations are more frequently seen in RCRC.^16^, ^17^ In our study, gene mutation is also more commonly seen in RCRC, and the proportion of KRAS and PIK3CA mutation is higher in RCRC. However, the frequency of BRAF mutation and MSI‐high is comparable between RCRC and LCRC. The mechanism of the molecular difference seen between two sides is unclear. Some researches claimed that the microbiota might contribute to the result. As the microbial flora diversity is different between RCRC and LCRC, the ability to produce short‐chain fatty acids, hydrolytic and reductive bacterial enzymes is different between the two sides, which may give rise to the difference in immunomodulatory, antiinflammatory properties.^18^, ^19^, ^20^ Moreover, it has been suggested that bacterial toxins and mutagenic CYP450 metabolites may be associated with the hypermutant status in RCRC.^16^

The RAS family is the most studied malignancy‐related gene in CRC. KRAS is the most frequently altered isoform. It activates the downstream cascades including the PIK3 pathways, which may affect the cell proliferation and differentiation. The incidence rate of KRAS mutation is reported to be 30%‐40% in CRC patients.^13^, ^21^ Most of the KRAS mutations occur in codon 12 and 13. In our study, the frequency of KRAS mutation is 36.1%, which is consistent with a previous study.^22^ KRAS mutation has a higher proportion in female and RCRC patients. Besides, there were two cases with concurrent KRAS codon 12 and 59 mutations, which suggests that concurrent mutations may occur in different codons in KRAS gene. The prevalence of NRAS mutation is 3.9%, which is consistent with the range of 2.2%‐7% as reported in other studies.^23^, ^24^, ^25^ Some researchers suggested that NRAS was more common in older patients.^10^ However, we did not find a remarkable correlation between NRAS mutation and clinicopathological features of CRC. As the least frequent mutation in RAS family, HRAS is not well‐studied so far. The HRAS mutation rate was 0.9% in our study, lower than the previous reported frequency of 3.3%.^26^ The prognostic value of RAS family is controversial. Many researches have focused on the correlation between KRAS mutation and prognosis of CRC. We found no significant difference of OS among KRAS, NRAS, HRAS mutation and wild type patients. In the KRAS mutation group, there is no remarkable variance of OS in G12D, G13D or other mutant sites, either (Figure 5B). It seems that the RAS mutation is not associated with prognosis of Chinese CRC patients. However, there are discrepancies in different studies. Tanaka et al reported that KRAS mutation was an independent risk factor associated with the prognosis of CRC.^27^ The RASCAL study investigated 2721 CRC patients and showed that KRAS mutation was associated with poor prognosis, and KRAS C12V mutation most remarkably associated with poor outcome.^28^ Moreover, Zlobec et al suggested that KRAS G12D mutation had more adverse outcomes than other KRAS mutations.^29^ However, Lee et al claimed that KRAS mutation was not associated with OS or disease‐free survival (DFS) of CRC patients drawn from TCGA and http://www.ncbi.nlm.nih.gov/geo/query/acc.cgi?acc=GSE39582 databases.^30^ Hence more studies are needed to clarify the prognostic value of the RAS family in CRC.

We found 35 cases with BRAF mutation, and V600E mutation accounts for 60% of them. The frequency of BRAF mutation in CRC reported in literature is around 4.7%‐20%.^31^ BRAF mutation is suggested to be associated with some clinical features such as right‐sided location, poor differentiation, peritoneal metastasis.^32^ No association between BRAF mutation and tumor location or metastatic sites was found, but we found that BRAF^V600E^ mutation was associated with right‐sided location. However, we only found that BRAF mutation was more likely seen in poor/undifferentiated tumors. Though KRAS/NRAS and BRAF are thought to be mutually exclusive,^7^, ^11^, ^12^ we found three cases with concurrent KRAS and BRAF mutation. None of these three cases involves V600E mutation. The mechanism through which KRAS and BRAF coexist is not clear. Some researchers think that tumor heterogeneity may play a role.^30^ It has been widely reported that there is a correlation between BRAF mutation and poor prognosis in CRC patients, especially in advanced stage.^33^ A pooled study of three randomized clinical trials showed worse outcome of OS for BRAF mutation patients, but the DFS and progression‐free survival (PFS) were comparable to those without BRAF mutation.^34^ The OS of patients with BRAF mutation also seems to be worse in our study (49.9 m vs 83.2 m). However, it did not reach statistical significance (*P* = .051). When we focused on BRAF V600E mutation only, we found that this subgroup had much poorer prognosis (24.8 m vs 83.2 m; *P* = .005). It seems that only Braf^V600E^ mutation, but not other subtypes, was associated with worse outcome in CRC. A study from Mayo Clinic also showed that ^Non‐V600^ BRAF mutations metastatic CRC defined a clinically distinct subtype of CRC with an excellent prognosis.^35^

Most cases did not test the MSI/MMR status in our study. In the cases with known MSI/MMR status, we found some cases with both MSI‐H/dMMR and KRAS/BRAF mutation, which means that they could coexist, concordant with previous reports.^36^ However, we did not find any correlation between MSI/MMR status and KRAS/BRAF mutations. Some literature shows that MSI status may not have a prognostic relevance in CRC,^37^ but Yang et al suggested that microsatellite stable (MSS) + BRAF mutation was a poor prognostic factor, while MSI + BRAF mutation was related to a moderate prognosis, and MSS/MSI + BRAF wild type was associated with a more favorable outcome.^38^ Murcia et al reported a similar result, which suggested that the combination of MSS, BRAF mutation and CIMP positive related to poor prognosis.^39^ As there are few researches on the relationship between MSI status and gene mutation, more attention on this issue is needed.

PIK3CA mutation is the second most frequently detected in our study. It is more common seen in well/moderate differentiated tumor and RCRC. It has been reported that PIK3CA is associated with other gene mutations, especially KRAS mutation.^24^ The association mainly attributed to exon 9 mutations, while we found no correlation between exon 20 and KRAS mutation. This phenomenon can be explained by a previous study which showed that PIK3CA exon 9 mutation depended on a Ras‐GTP pattern, whereas exon 20 mutation did not involve Ras.^40^ The prognostic value of PIK3CA mutation is controversial. Some studies showed that PIK3CA mutation was associated with shorter PFS and/or OS.^24^, ^41^ Ogino et al suggested that PIK3CA mutations were a poor prognostic factor in stage I‐III CRC patients.^42^ However, we did not find PIK3CA mutation associated with poor outcome. More studies are needed to clarify the role of PIK3CA mutation in CRC.

Our study has some limitations.. First, as a retrospective research, selection bias inevitably exists. Most cases were diagnosed at advanced stage, and few patients with early stages were included. Second, some patients may be lost to follow‐up, hence their medical record and data may be lost. Therefore, more researches, especially perspective ones, are needed to clarify the role of gene mutation in the prognosis of CRC.

## 5 CONCLUSION

Compared to the previous study that we published,^43^ we expanded the sample size, described a more comprehensive picture of the gene mutation profile in Chinese CRC patients, and investigated the prognosis value of the most commonly mutated genes, such as RAS family, BRAF, and PIK3CA. Other rare mutations, such as AKT1, KIT, FGFR1, FGFR3, FLT3, CDK, ERBB2, ABL1, MET, RET and PDGFRA, were extremely rare in CRC. Knowledge of the gene mutation patterns may help to investigating their roles in CRC. It may also gives clues in the research and development of new drugs.

## CONFLICT OF INTEREST

None declared.

## CONSENT FOR PUBLICATION

All the authors have read and approved the final manuscript.

## Data Availability

Data are available on request.
