# Supplementary figures and images for: Gene mutation profiling in Chinese colorectal cancer patients and its association with clinicopathological characteristics and prognosis
Source: Cancer Med. 2019 Nov 28;9(2):745–56. doi: 10.1002/cam4.2727 (PMC6970031; doi:10.1002/cam4.2727)

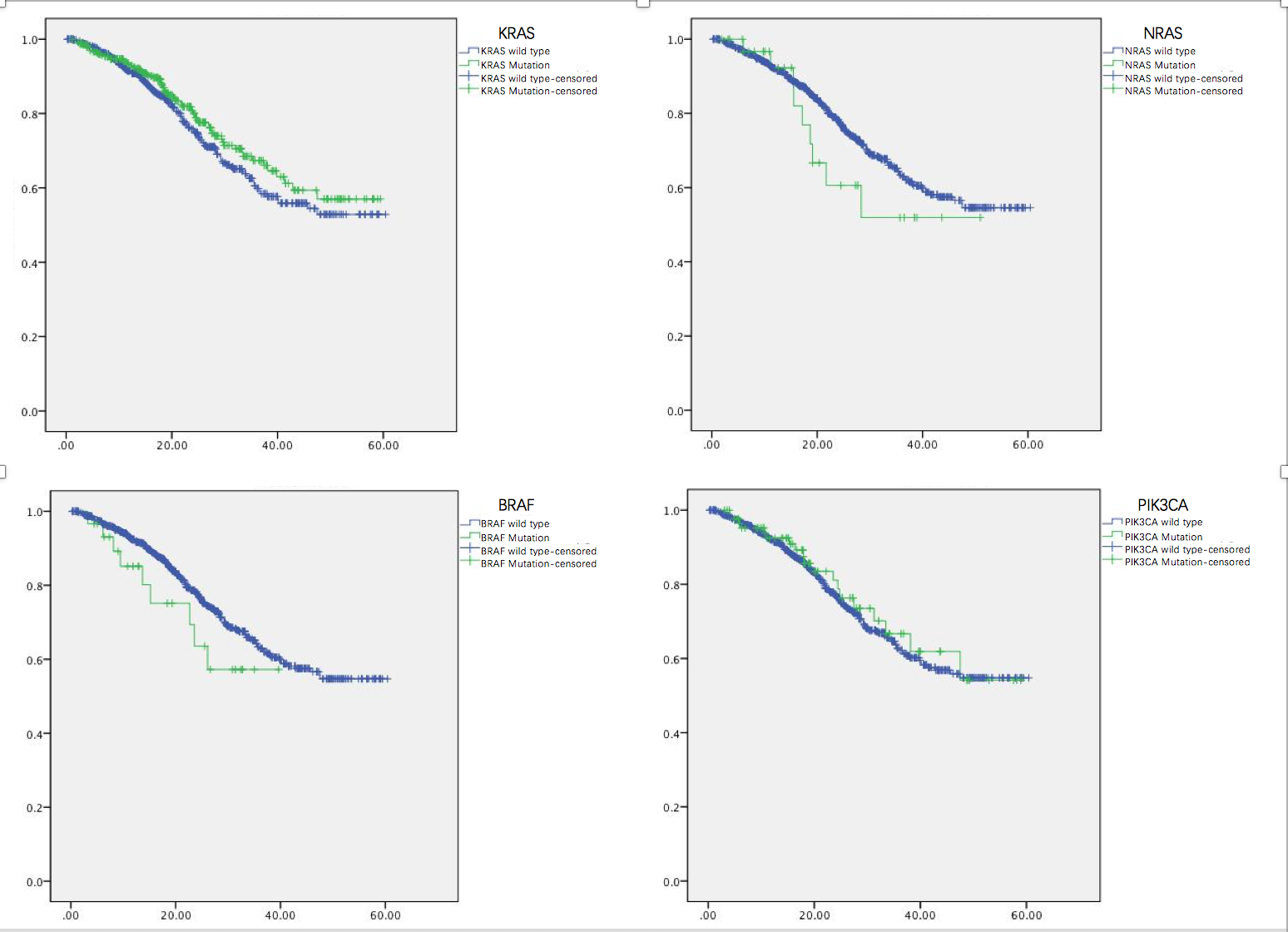

Supplement: Supplementary file 1 [file CAM4-9-745-s001.tif]

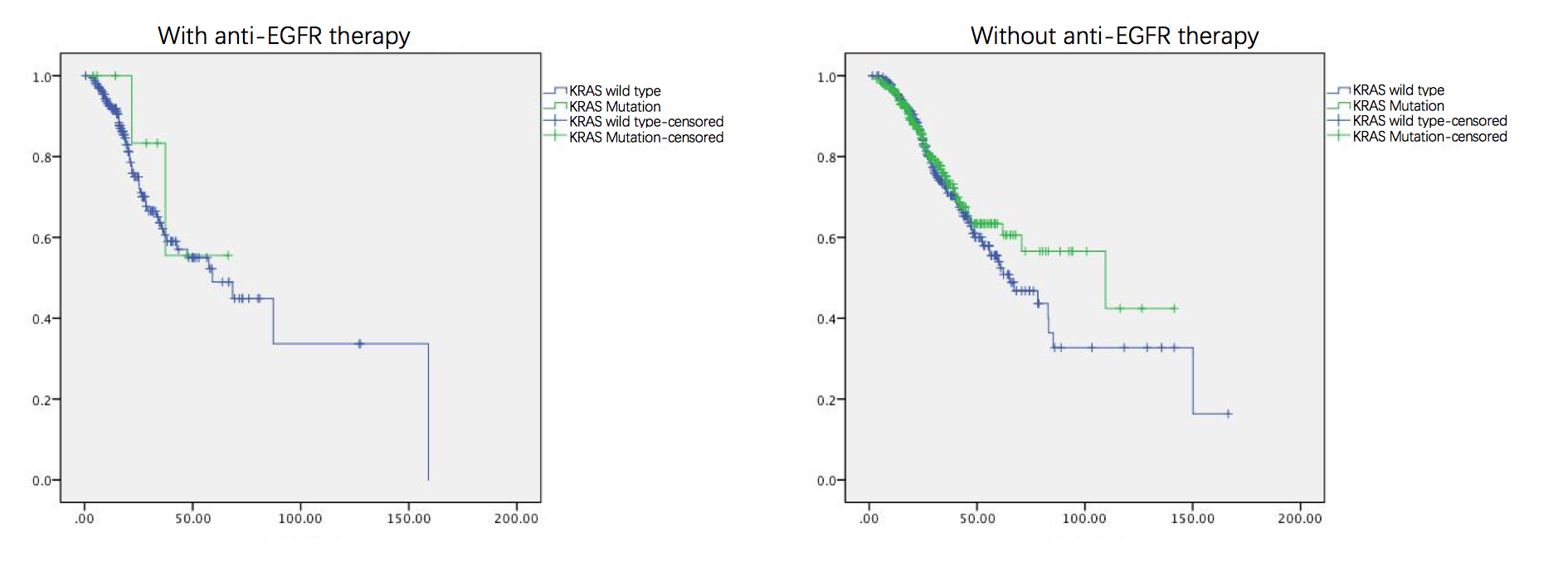

Supplement: Supplementary file 2 [file CAM4-9-745-s002.tif]

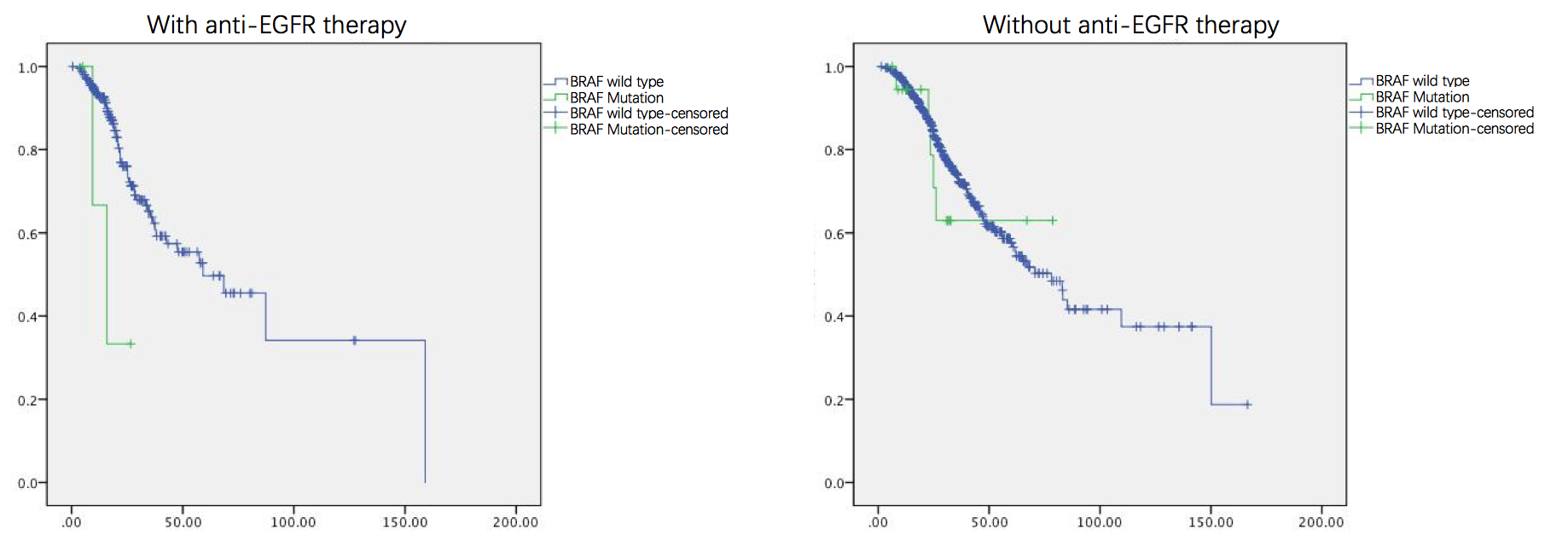

Supplement: Supplementary file 3 [file CAM4-9-745-s003.tif]
