# Supplementary material for: Gene mutation profiling in Chinese colorectal cancer patients and its association with clinicopathological characteristics and prognosis
Source: Cancer Med. 2019 Nov 28;9(2):745–56. doi: 10.1002/cam4.2727 (PMC6970031; doi:10.1002/cam4.2727)
Supplement: Supplementary file 4 [file CAM4-9-745-s004.docx]

| Supplementary Table 1: Mutations included in the OncoCarta panel | | | |
| --- | --- | --- | --- |
| Gene | NCBI Reference Sequence | Locations | Mutation Detection |
| ABL1 | NM_005157 | 9q34.1 | G250E, Q252H, Y253H, Y253F, E255K, E255V,D276G, F311L, T315I, F317L, M351T, E355G,F359V, H396R |
| AKT1 | NM_005163 | 14q32.32 | V461L, P388T, L357T, E319G, V167A, Q43X,E17K |
| AKT2 | NM_001626 | 9q13.1-q13.2 | S302G, R371H |
| BRAF | NM_004333 | 7q34 | G464R, G464V/E, G466R, F468C, G469S, G469E,G469A, G469V, G469R, G469R, G596R,L597S, L597R, L597Q, L597V, T599I,D594V/G, F595L,V600E, V600K, V600R, V600L, K601N, K601E |
| CDK4 | NM_000075 | 12q14 | R24C, R24H |
| EGFR | NM_005228 | 7p21 | R108K, T263P, A289V, G598V, E709K/H, E709A/G/V, G719S/C, G719A,V769_D770insASV, V769_D770insCV, V769_D770insASV, V769_D770insCV,M766_A767insAI, S768I,D770_N771>AGG/V769_D770insASV/V769_D770insASV, D770_N771insG,N771_P772>SVDNR, P772_H773insV, H773_V774insNPH/PH/H, V774_C775insHV,H773>NPY,T790M, L858R, L861Q, E746_T751del,E746_A750del, E746_T751del, E746_T751del,S752D, L747_E749del, L747_T750del,L747_S752del, L747_T751del, L747_S752del,P753S(combined), A750P, T751A, T751P, T751I, S752I/F,S752_I759del, L747_Q ins, E746_T751del, I ins(combined), E746_A750del, T751A (combined),L747_E749del, A750P (combined), L747_T750del,P ins (combined), L747_S752del, Q ins (combined) |
| ERBB2 | NM_004448 | 17q21.1 | L755P, G776S/LC, G776VC,A775_G776insYVMA, P780_Y781insGSP,P780_Y781insGSP, S779_P780insVGS |
| FGFR1 | NM_023110 | 8p11.2-p11.1 | S125L, P252T |
| FGFR3 | NM_000142 | 4p16.3 | G370C, Y373C, A391E, K650Q/E, K650T/M |
| FLT3 | NM_004119 | 13q12 | I836del, D835H/Y |
| HRAS | NM_005343 | 11p15.5 | G12V/D, G13C/R/S, Q61H/H, Q61L/R/P, Q61K |
| JAK2 | NM_004972 | 9p24 | V617F |
| KIT | NM_000222 | 4q11-q12 | D52N, Y503_F504insAY, W557R/R/G, V559D/A/G,V559I, V560D/G, K550_K558del, K558_V560del,K558_E562del, V559del, V559_V560del, V560del,Y570_L576del, E561K, L576P, P585P, D579del,K642E, D816V, D816H/Y, V825A, E839K, M552L,Y568D, F584S, P551_V555del, Y553_Q556del |
| KRAS | NM_033360 | 12p12.1 | G12C, G12R, G12S, G12V, G12D, G12A, G12F,G13V/D, A59T, Q61E/K, Q61L/R/P, Q61H |
| MET | NM_000245 | 7q31 | R970C, T992I, Y1230C, Y1235D, M1250T |
| NRAS | NM_002524 | 1p13.2 | G12V/A/D, G12C/R/S, G13V/A/D, G13C/R/S, A18T,Q61L/R/P, Q61H, Q61E/K |
| PDGFRA | NM_006206 | 7p22 | V561D, T674I, F808L, D846Y, N870S, D1071N,D842_H845del, I843_D846del, S566_E571>K,I843_S847>T, D842V |
| PIK3CA | NM_006218 | 3q26.3 | R38H,R88Q, N345K, C420R, P539R, E542K, E545K,Q546K, H701P, C901F,M1043I, H1047R/L, H1047Y |
| RET | NM_020975 | 10q11.2 | C634R, C634W, C634Y, E632_L633del, M918T,A664D |

| Supplementary Table 2: Summary of mutations identifies in 1190 patients with colorectal cancer | | | | |  |  |  |
| --- | --- | --- | --- | --- | --- | --- | --- |
|  | Mutation | |  | N% (1190) |  |  |  |
| Gene | Mutation | | Cases |  |  |  |  |
| KRAS | G12C | | 43 | 3.6 |  |  |  |
|  | G12R | | 2 | 0.2 |  |  |  |
|  | G12S | | 26 | 2.2 |  |  |  |
|  | G12V | | 78 | 6.6 |  |  |  |
|  | G12D | | 148 | 12.4 |  |  |  |
|  | G12A | | 15 | 1.3 |  |  |  |
|  | G13D | | 85 | 7.1 |  |  |  |
|  | A59T | | 3 | 0.3 |  |  |  |
|  | Q61K | | 3 | 0.3 |  |  |  |
|  | Q61L | | 7 | 0.6 |  |  |  |
|  | Q61R | | 5 | 0.4 |  |  |  |
|  | Q61H | | 12 | 1.0 |  |  |  |
|  | G12D&A59T | | 2 | 0.2 |  |  |  |
|  | total | | 429 | 36.1 |  |  |  |
| NRAS | G12V | | 2 | 0.2 |  |  |  |
|  | G12D | | 9 | 0.8 |  |  |  |
|  | G12S | | 7 | 0.6 |  |  |  |
|  | G13V | | 1 | 0.1 |  |  |  |
|  | G13D | | 1 | 0.1 |  |  |  |
|  | G13R | | 2 | 0.2 |  |  |  |
|  | G13S | | 2 | 0.2 |  |  |  |
|  | A18T | | 1 | 0.1 |  |  |  |
|  | Q61L | | 6 | 0.5 |  |  |  |
|  | Q61R | | 7 | 0.6 |  |  |  |
|  | Q61K | | 8 | 0.7 |  |  |  |
|  | G12S&Q61R | | 1 | 0.1 |  |  |  |
|  | total | | 47 | 3.9 |  |  |  |
| HRAS | G12D | | 1 | 0.1 |  |  |  |
|  | G13S | | 9 | 0.8 |  |  |  |
|  | Q61R | | 1 | 0.1 |  |  |  |
|  | total | | 11 | 0.9 |  |  |  |
| BRAF | G464V | | 2 | 0.2 |  |  |  |
|  | G464E | | 2 | 0.2 |  |  |  |
|  | G469E | | 2 | 0.2 |  |  |  |
|  | G469A | | 2 | 0.2 |  |  |  |
|  | G469R | | 2 | 0.2 |  |  |  |
|  | D594V/G | | 1 | 0.1 |  |  |  |
|  | V600E | | 21 | 1.8 |  |  |  |
|  | V600K | | 2 | 0.2 |  |  |  |
|  | K601E | | 1 | 0.1 |  |  |  |
|  | total | | 35 | 2.9 |  |  |  |
| PIK3CA | R38H | | 1 | 0.1 |  |  |  |
|  | R88Q | | 8 | 0.7 |  |  |  |
|  | N345K | | 1 | 0.1 |  |  |  |
|  | C420R | | 8 | 0.7 |  |  |  |
|  | P539R | | 1 | 0.1 |  |  |  |
|  | E542K | | 24 | 2.0 |  |  |  |
|  | E545K | | 28 | 2.4 |  |  |  |
|  | Q546K | | 6 | 0.5 |  |  |  |
|  | M1043I | | 6 | 0.5 |  |  |  |
|  | H1047R | | 28 | 2.4 |  |  |  |
|  | H1047L | | 9 | 0.8 |  |  |  |
|  | C420R&E545K | | 1 | 0.1 |  |  |  |
|  | total | | 121 | 10.2 |  |  |  |
| EGFR | G598V | | 1 | 0.1 |  |  |  |
|  | G719S | | 1 | 0.1 |  |  |  |
|  | E709K | | 1 | 0.1 |  |  |  |
|  | E746_S752>I | | 1 | 0.1 |  |  |  |
|  | L747_S752del,P753S(combined) | | 3 | 0.3 |  |  |  |
|  | H773_V774insNPH | | 2 | 0.2 |  |  |  |
|  | L858R | | 2 | 0.2 |  |  |  |
|  | total | | 11 | 0.9 |  |  |  |
| KIT | D52N | | 2 | 0.2 |  |  |  |
|  | K558_V560del | | 1 | 0.1 |  |  |  |
|  | K558_E562del | | 2 | 0.2 |  |  |  |
|  | L576P | | 2 | 0.2 |  |  |  |
|  | total | | 7 | 0.6 |  |  |  |
| AKT1 | E17K | | 8 | 0.7 |  |  |  |
|  | Q43X | | 1 | 0.1 |  |  |  |
|  | V461L | | 1 | 0.1 |  |  |  |
|  | total | | 10 | 0.8 |  |  |  |
| ERBB2 | G776S | | 2 | 0.2 |  |  |  |
|  | G776VC | | 1 | 0.1 |  |  |  |
|  | total | | 3 | 0.3 |  |  |  |
| FGFR3 | G370C | | 2 | 0.2 |  |  |  |
|  | A391E | | 2 | 0.2 |  |  |  |
|  | total | | 4 | 0.3 |  |  |  |
| FLT3 | D835Y | | 1 | 0.1 |  |  |  |
|  | I836del | | 4 | 0.3 |  |  |  |
|  | total | | 5 | 0.4 |  |  |  |
| ABL1 | T315I | | 3 | 0.3 |  |  |  |
| CDK | R24C | | 5 | 0.4 |  |  |  |
| FGFR1 | S125L | | 7 | 0.6 |  |  |  |
| MET | T992I | | 1 | 0.1 |  |  |  |
| PDGFRA | D842V | | 1 | 0.1 |  |  |  |
| RET | C634Y | | 1 | 0.1 |  |  |  |
| Supplementary Table 3: Frequency of mutation in RAS family in patients with colorectal cancer | | | | | |  |  |
| Genes | | Mutation | | | | Cases with mutation (%) | |
| Total cases with RAS mutation | |  | | | | 476 | 40.0 |
| Total cases with KRAS mutation | |  | | | | 429 | 36.1 |
| KRAS codon 12 | | G12C | | | | 43 | 3.6 |
|  | | G12R | | | | 2 | 0.2 |
|  | | G12S | | | | 26 | 2.2 |
|  | | G12V | | | | 78 | 6.6 |
|  | | G12D | | | | 148 | 12.4 |
|  | | G12A | | | | 15 | 1.3 |
| KRAS codon 13 | | G13D | | | | 85 | 7.1 |
| KRAS codon 59 | | A59T | | | | 3 | 0.3 |
| KRAS codon 61 | | Q61K | | | | 3 | 0.3 |
|  | | Q61L | | | | 7 | 0.6 |
|  | | Q61R | | | | 5 | 0.4 |
|  | | Q61H | | | | 12 | 1.0 |
| KRAS codon 12&59 | | G12D&A59T | | | | 2 | 0.2 |
| Total cases with NRAS mutation | |  | | | | 47 | 3.9 |
| NRAS codon 12 | | G12V | | | | 2 | 0.2 |
|  | | G12D | | | | 9 | 0.8 |
|  | | G12S | | | | 7 | 0.6 |
| NRAS codon 13 | | G13V | | | | 1 | 0.1 |
|  | | G13D | | | | 1 | 0.1 |
|  | | G13R | | | | 2 | 0.2 |
|  | | G13S | | | | 2 | 0.2 |
| NRAS codon 18 | | A18T | | | | 1 | 0.1 |
| NRAS codon 61 | | Q61L | | | | 6 | 0.5 |
|  | | Q61R | | | | 7 | 0.6 |
|  | | Q61K | | | | 8 | 0.7 |
| KRAS codon 12&61 | | G12S&Q61R | | | | 1 | 0.1 |
| Total cases with HRAS mutation | |  | | | | 11 | 0.9 |
| HRAS codon 12 | | G12D | | | | 1 | 0.1 |
| HRAS codon 13 | | G13S | | | | 9 | 0.8 |
| HRAS codon 61 | | Q61R | | | | 1 | 0.1 |
